# The Hospital Saturday Fund Disagreement

**Published:** 1891-02-21

**Authors:** 


### Passing Topics.

### THE HOSPITAL SATURDAY FUND DISAGREEMENT.

Recent occurrences seem to have supplied the critics of the Hospital Saturday Fund with fresh matter upon which to exercise their reflective powers. An expenditure of no less than £1,365 upon office salaries looks at first sight little justified in connection with the administration of a fund of no greater magnitude than this, and must swell the total of management expenses, all told, to an unconscionable percentage upon the amount distributed.

A remarkable fact is that no individual officer obtains a stipend which, if he be the right man in the right place, the most economical can denounce as excessive. It is the grand aggregate of personalities, nine men and a boy, which conveys to the mind a sense of almost ludicrous disproportion and extravagance. Moreover, if we omit two men and the boy, there is an excess of equality, in actual if not in nominal position, among the remainder which augurs ill for discipline and smooth working, and it is difficult to avoid some sympathy with the Committee which reported that "the work of the Fund had been seriously hampered by the present system of divided authority, and it was essential that there should be in future only one Secretary." There is no doubt this is a common-sense view, even though we take exception to the way in which it is proposed to give immediate effect to it. Assuming the "General Secretary" an the "Organising Secretary" to be efficient officers and worthy of the confidence of their Committee, it seems a little hard they should be called upon to resign their positions, virtually without notice, and the severity of the proceeding is not mitigated but rather aggravated by the suggestion that they may take an equal chance with all other applicants for the one secretaryship it is proposed to create.

Reforms are none the less effectual for being carried out with some delicacy of touch, and opportunity should never be given to associate reasonable measures with injustice to those chiefly concerned. It is astonishing how ready some people are to justify the means by the end they have in view, but, put plainly, it is no more right to mulct the officials of a charitable fund of what is customarily due than to wrong workers in any other field. No doubt the belief that it was proposed to mete out less than justice to faithful servants induced the delegates of the Fund to throw over the recommendations of the Committee.

When we come to consider the matter upon its merits alone, few will question the need for reorganization, and especially if, as is somewhat freely asserted, there has been a lack of efficiency; but while admitting that the total expenditure upon salaries appears very great, it is right to add that the disproportion is probably greater in regard to the amount collected than to the labour incidental to and inseparable from the collection. The result is obvious and palpable: the time and labour expended are hidden away, and can only be realised by those who have experienced the difficulties in the way of getting together a fund of £10,000.

We have never professed any great admiration for the methods of the Saturday Fund authorities, nor are we disposed to abate our former criticisms; but although we still consider the aggregate a sorry recognition of the debt the working classes owe to the hospitals, and regard the claims for patronage in connection with its distribution as altogether ridiculous, we are profoundly sensible of the labour involved in the collection of a fund of which the component items are so minute. The offerings of the working classes are not likely to be one whit more spontaneously made than those of the classes in possession of greater resources, and the process of coaxing the pence into a respectable total is necessarily long and tedious. It is only fair to both Committee and officers to remember that fact when we attempt to reckon up the cost of raising the Hospital Saturday Fund.

Agricola.

